# The Arsenicai Commission

**Published:** 1903-12-12

**Authors:** 


The Hospital.

A Journal of

The Medical Sciences and Hospital Administration.

Vol. XXXV.—No. 898. DECEMBER 12, 1903.

### The Arsenical Commission.

In more than one recent article we have pointed out the importance of such an education of the poor as might enable them to decide correctly upon the respective claims to their patronage of the various kinds of food which are pressed upon their notice by purveyors; or, failing such education, upon the importance of some official supervision of advertised foods and drinks, with a view either to the maintenance of some fixed standard of purity and nutritive value, or to the display of storm warnings in the cases in which such standards were not likely to be either attained or aimed at. Our observations in this direction have since been remarkably confirmed by the final report of the Royal Commission on Arsenical Poisoning; a commission which, as our readers will remember, was appointed in February, 1901, in consequence of the discovery, by Dr. E. S. Reynolds, of Manchester, that numerous cases of illness which had occurred in that city and its neighbourhood were due to chronic arsenical poisoning, and to the further discovery that the poisoning had been effected through the agency of beer, into which arsenic had been introduced by the sulphuric acid employed in the manufacture of brewing sugars or glucose. The poisoning in question was, indeed, epidemic in Manchester, Salford, and other places in the Northern and North Midland districts of England; and the Commission was soon able to present an interim report tracing it to its causes, and pointing to measures by which the immediate plague might be stayed. Large quantities of contaminated beer were suffered to run into the sewers, large quantities of contaminated artificial sugars and syrups were either destroyed or sold subject to undertakings that they were only to be used for purposes unconnected with food, and the epidemic of neuritis which had been described and treated as "alcoholic" soon ceased to be a conspicuous feature in the sanitary history of the localities chiefly affected. Notwithstanding so much improvement, the Commission, under the chairmanship of Lord Kelvin, and composed of Sir William Hart Dyke, Professor Thorpe, Mr. Cosmo Bonsor, Sir William Church, and Dr. Whitelegge, found it expedient to continue their inquiries, which had early convinced them that the contamination of beer by arsenical sugar by no means exhausted the possibilities of what may be humourously called "accidental" poisoning by arsenic contained in matters of common use; and the result of their decision has been the issue of a final report, in which the causes of arsenical contamination are fully discussed with reference to a great variety of substances, and in which the possibilities of legislative protection are carefully considered. The magnitude of the whole question is shown by the statement that what was called the Manchester epidemic was known to have affected 6,000 persons, and was believed to have affected many more; while the ascertained deaths were over 100, leaving an indefinite number registered as from various causes, but as to which the influence of arsenic could be reasonably suspected.

Perhaps the most remarkable result of the inquiry was the discovery that, although the bulk of cases in the Manchester epidemic were clearly due to contaminated sugar, yet illness of a similar kind had prevailed extensively in various localities in which contaminated sugar had not been used by brewers, and that, when its arsenical character was no longer in doubt, it was without much difficulty traced to the contamination of malt by the use of arsenical gas coke in the drying kilns. Certain samples of such malt contained as much as $\frac{1}{60}$th of a grain of arsenic to the pound; and beer brewed from malt prepared in a similar manner was found to contain $\frac{1}{16}$th of a grain of arsenic to the gallon. After fully discussing the questions hence arising, the report goes on to enumerate other methods by which arsenic may be introduced into common forms of food, and the list of foods liable to be so affected includes beer, golden syrup and treacle, foods containing glucose, vinegar, Demerara sugar, various extracts of malt, manufactured either for sale to invalids or for use by bakers, "prepared" and "infant" foods under a variety of names, yeast, cakes and foods to which certain colouring matters or preservatives have been added. Mr. Hehner has found from $\frac{1}{20}$th to $\frac{1}{25}$th of a grain of arsenic per lb. in a so-called "chocolate powder" sold in London at a low price, and largely composed of an arsenicated oxide of iron.

The means suggested by the Commission for the better protection of purchasers appear to be at once moderate and practicable, and there can be no doubt as to the urgency of the requirement. It is proposed to place the administration of the Food and

Drugs Acts largely under the direction of the Local Government Board, assisted by the advice of a special officer with suitable scientific knowledge. It is suggested that this officer should have the duty not only of collecting information from public analysts and other local officers, and of advising how work under the Acts may be conducted on satisfactory lines, but also of making inspections when necessary, and of instituting inquiries as to conditions of food manufacture. He should also obtain all information possible regarding the manufacture of food stuffs imported from abroad, and should report annually. By such means, in the opinion of the Commission, the Local Government Board would be able not only to advise and direct the work of local authorities in a way not hitherto attempted, but also to make suggestions which would be of material assistance to manufacturers anxious to secure the purity of their products. We do not know to what extent "anxiety" of this kind can be said to exist at present; but it might, no doubt, be stimulated into activity by well-directed applications of the law.

